# An evaluation of *OPTC* and *EPYC* as candidate genes for high myopia

**Published:** 2009-10-15

**Authors:** Panfeng Wang, Shiqiang Li, Xueshan Xiao, Xiangming Guo, Qingjiong Zhang

**Affiliations:** State Key Laboratory of Ophthalmology, Zhongshan Ophthalmic Center, Sun Yat-sen University, Guangzhou, P. R. China

## Abstract

**Purpose:**

The small leucine-rich repeat proteins (SLRPs) are involved in organizing the collagen fibrils of the sclera and vitreous. The shape of the eyeball is determined by the sclera and vitreous, so defects in SLRP family members may contribute to myopia. The purpose of this study was to test whether mutations in the two members of the class III SLRPs, opticin (*OPTC*) and dermatan sulfate proteoglycan 3 (*EPYC*), are responsible for high myopia.

**Methods:**

DNA was prepared from venous leukocytes of 93 patients with high myopia (refraction of spherical equivalent ≤-6.00D) and 96 controls (refraction of spherical equivalent between -0.50D and +1.00D). The coding regions and adjacent intronic sequences of *OPTC* and *EPYC* were amplified by the polymerase chain reaction (PCR), and the products were then analyzed by cycle sequencing. The detected variations were further evaluated in normal controls and available family members by a heteroduplex-single strand conformation polymorphism (heteroduplex-SSCP) analysis or sequencing.

**Results:**

Two substitutions in *OPTC,* including c.491G>T and c.803T>C, were identified. The c.491G>T mutation (p.Arg164Leu), a novel heterozygous variation, was detected in one of the 93 patients but in none of the 96 controls. The c.803T>C mutation (p.Pro267Leu), a known polymorphism, was detected in 22 of the 93 patients and in 15 of 48 controls. No variation was observed in *EPYC*.

**Conclusions:**

Only one novel variation in *OPTC* was detected in a Chinese patient with high myopia. Our results imply that *OPTC* and *EPYC* are unlikely to play a major role in high myopia.

## Introduction

Myopia is the most common human vision impairment all over the world, especially in Chinese people living in urban/suburban areas [1-6]. High myopia, an extreme form of myopia with refraction less than -6.0D (diopters), is a common cause of irreversible blindness due to its association with retinal degeneration and detachment, glaucoma, and other abnormalities. The exact molecular basis of high myopia and the genes that cause a predisposition to this disorder are still unknown, although abundant evidence has demonstrated that genetic factors play an important role in the development of high myopia [7].

The shape of the eye is largely determined by the sclera and the vitreous that are composed of abundant extracellular matrix (ECM) [8]. Abnormalities due to mutations in several ECM proteins or proteoglycans, such as *COL2A1* and *COL11A1* in Stickler syndrome and *fibrillin-1* in Marfan syndrome, have been associated with syndromic high myopia [9,10]. The small leucine-rich repeat proteins (SLRPs), members of the ECM family, play important roles in organizing collagen fibrils [11]. Experiments in *SLRP* knockout mice have shown that a wide array of defects are caused by abnormal collagen fibrillogenesis, resulting in a longer eye axial length and high myopia [12]. Therefore, it would be reasonable to determine whether there are mutations in these SLRPs in human subjects with high myopia.

The *OPTC* gene (gene ID: 26254; OMIM 605127), located in chromosome 1q32.1, is a member of the class III SLRPs [13], and the OPTC protein is widely distributed in ocular tissue, including the cornea, iris, trabecular meshwork, ciliary body, retina, and optic nerve. *OPTC* is specifically expressed in the vitreous humor where the protein plays a key role in maintaining the gel structure of the vitreous humor [14-16]. The dermatan sulfate proteoglycan 3 gene (*DSPG3/EPYC*; GENE ID: 1833; OMIM 601657), located in chromosome 12q21 where MYP3 (OMIM 603221) mapped [17,18], is another member of the class III SLRPs [19]. The EPYC protein is predominantly expressed in cartilage [20,21] and is important for fibrillogenesis through the regulation of collagen fibrils.

In this study, the coding exons and the adjacent non-coding regions of *OPTC* and *EPYC* were analyzed in 93 Chinese patients with high myopia.

## Methods

### Subjects

The procedure for collecting the subjects and obtaining informed consent was the same as previously described [22]. The study was carried out following the tenets of the Declaration of Helsinki and was approved by the Institute Review Board of the Zhongshan Ophthalmic Center. Informed consent was obtained from the participating subjects before the study. Ophthalmological examinations were performed by ophthalmologists of the Zhongshan Ophthalmic Center (Q.Z. and X.G.). The recruitment of subjects as same as we described previously [23].

### Mutation analysis

**Figure 1:**
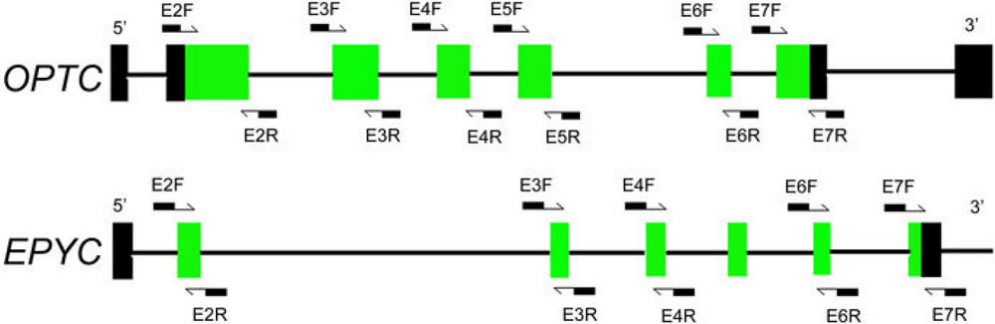
The exon-intron structures of *OPTC* and *EYPC*. The black rectangles represent the untranslated region and the green rectangles represent the coding region.

Genomic DNA was prepared from peripheral leukocytes as described previously [24]. Table 1 lists the primers used to amplify the coding exons and adjacent introns of *OPTC* (NCBI human genome build 36.3, NC_000001.10 for genomic DNA, NM_ 014359.3 for mRNA and NP_ 055174.1 for protein) and *EPYC* (NCBI human genome build 36.2, NC_000012.11 for genomic DNA, NM_004950.4 for mRNA and NP_004941.2 for protein; Figure 1). The nucleotide sequences of *OPTC* and *EPYC* were determined by cycle sequencing. The sequencing results from patients as well as the *OPTC* (NT_004487.19) and *EPYC* (NT_029419.12) consensus sequences were compared to identify variations. The variations were confirmed by bi-directional sequencing. The description of the variations followed the nomenclature recommended by the Human Genomic Variation Society (HGVS). The mutation we detected was further evaluated in 96 normal controls by single-strand conformational polymorphism (SSCP) using an extra pair of primers (Table 1), using the method we described previously [24]. The SIFT program (Sorting Intolerant From Tolerant) was used to predict whether an amino acid substitution was likely to affect the protein function [26].

**Table 1:** Primers used for the amplification and sequencing of *OPTC* and *EPYC*.

**Gene**	**Exon**	**Direction**	**Primer sequence**	**Size of PCR product (bp)**	**Annealing temperature (°C)**
*OPTC*	E2	F	5'-CGAGCCATTCCCACAACACT-3'	439	64
		R	5'-GCTGGAGACACCCGACTTT-3'		
	E3	F	5'-TTCCCTCCTCCTCCTCCACA-3'	387	62
		R	5'-GTAGCCCTGACCCTCCAT-3'		
	E4	F	5'-TGTACATGGTGCCCGTCAGT-3'	425	62
		R	5'-GAGAGCCAACAGGAAAGAGC-3'		
	E5	F	5'-AGGTGGAGAGCTGGTTAGAA-3'	433	64
		R	5'-GGTGGTGGTGGAGGTGAGTG-3'		
	E6	F	5'-GCGCATGGCTGAGGCTAAG-3'	346	62
		R	5'-CAATCAAGGAGACCCCACAG-3'		
	E7	F	5'-TTGGCCCTAGATGCTTCAGT-3'	425	62
		R	5'-ATTAATGCCTGGGCTAAAGA-3'		
	SSCP	F	5'-CTGCGTGTGCCTCGGTTCCTCT-3'	180	64
		R	5'-ATGCCTGCCCCCACTCCTCTTT-3'		
*EPYC*	E2	F	5'-ACATCTGGCAGCAATGGTC-3'	538	60
		R	5'-TCCGGTATTAACCTCCAAAAG-3'		
	E3	F	5'-CAAGCAGGATGGTCAAACTAT-3'	451	62
		R	5'-TGCTTCAAACCTAGCCTATTC-3'		
	E4	F	5'-CTTGGTTAGTGGGAGACTCTA-3'	371	62
		R	5'-AACCAGGCTTTGGCATTCAA-3'		
	E5-1*	F	5'-ATTAACTGTGCATTGCTAAAT-3'	473	44-68
		R	5'-AAGCCTGGGTGACAGAGTGA-3'		
	E5-2*	F	5'-TAATGCCTGTATCTTTGACTAT-3'	630	44-68
		R	5'-AATTTATTTTCCCTACACGA-3'		
	E5-3*	F	5'-GTTGCTTTATGATGGATGAATG-3'	979	44-46
		R	5'-CCTAGGAAATATGGCGAAACC-3'		
	E5-4 [25]	F	5'-CGTGGTGTACCAATTTCTTTGCT-3'	579	44-68
		R	5'-ACTCAAGCCTGGGTGACAGAGTG-3'		
	E6	F	5'-AAAACTAAGCTGCCTCAACT-3'	353	60
		R	5'-AACATGCCATTACAGACAAA-3'		
	E7	F	5'-TTACCCCCATTGCTTAGAAA-3'	433	60
		R	5'-GAACACAATCTCAAAGTATCA-3'		

The asterisk indicates that in order to amplify exon 5 of *EPYC*, the PCR conditions, including DMSO concentration, and the enzymes used (rTaq, LATaq, hotMaster, and AmpiTaq Gold), were varied.

## Results

**Figure 2:**
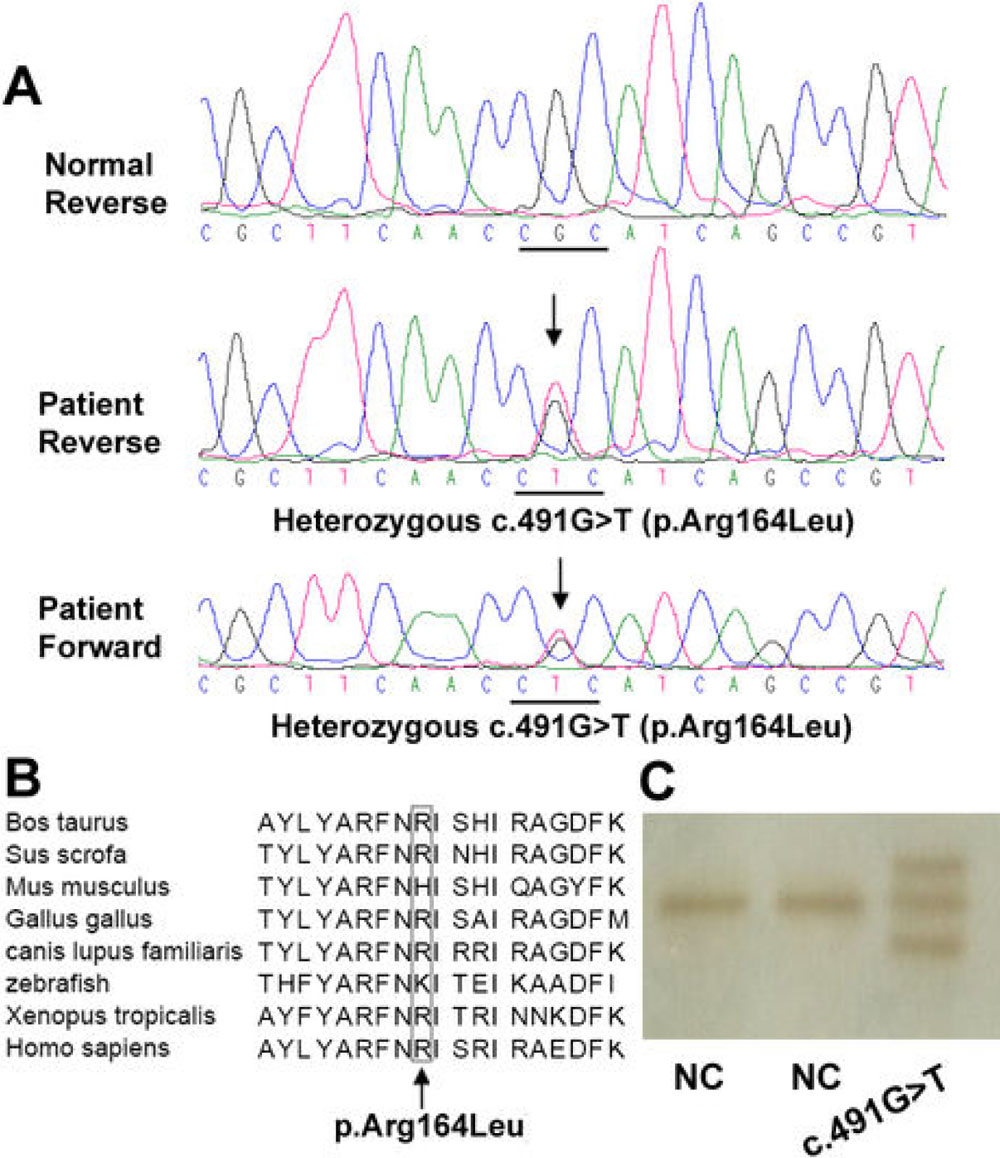
The novel variation identified in *OPTC*. **A**: The c.491G>T variant (indicated by an arrow) was sequenced in two directions and was compared to the wild type sequences. **B**: The missense variation p.Arg164Leu (c.491G>T; indicated by an arrow) changed a highly conserved residue from arginine to leucine. **C**: Variant c.491G>T (p.Arg164Leu) was not observed in the 96 normal controls by the heteroduplex-SSCP analysis.

After sequencing the *OPTC* gene from 93 high myopia patients, two missense variations, c.491G>T and c.803T>C, were identified. The c.491G>T mutation is a novel heterozygous variation in which an arginine (a charged amino acid) would be replaced by a leucine (an aliphatic, hydrophobic amino acid) in the encoded protein (p.Arg164Leu; Figure 2A), with a residue weight of -2 (Blosum 62). The arginine at position 164 of *OPTC* is conserved among the six orthologs from *Homo sapiens* (NP_055174), *Bos Taurus* (NP_991339), *Canis lupus familiaris* (NP_001003056), *Gallus gallus* (NP_989804), *Sus scrofa* (NP_999173), and *Xenopus tropicalis* (NP_001016499; Figure 2B), but is not conserved in two orthologs from *Mus Musculus* (NP_473417) and Zebrafish (NP_001003583). The SIFT analysis predicted that the substitution at position 164 from arginine to leucine would to be tolerated with a score of 0.17. Substitutions with a score less than 0.05 are predicted to affect protein function [26]. This novel variant was identified only in a single patient, but not in the 96 controls, by SSCP analysis (Figure 2C). However, the c.803T>C mutation (p.Pro267Leu), a known polymorphism, was detected in 22 of the 93 patients with high myopia (18 heterozygous and 4 homozygous) as well as in 15 of the 48 controls (13 heterozygous and 2 homozygous).

No variation was identified in exons 2, 3, 4, 6, and 7 of *EPYC*. Our attempts to amplify part or all of exon 5 of *EPYC* failed, possibly due to special DNA structures in the region, such as those caused by a high AT content.

## Discussion

*OPTC* is widely expressed in ocular tissue and was screened as a candidate gene in patients with high myopia, glaucoma, and age-related macular degeneration [27-30]. Recently, mutations in *OPTC* were suggested to be genetic risk factors underlying the pathogenesis of high myopia, where five variations were identified in five of 125 Caucasian patients with high myopia [30]. Co-segregation analyses were carried out in four families, where three variants (p.Arg325Trp, p.Arg330His, and p.Gly329Ser) were also present in relatives with normal refraction or moderate myopia. The p.Thr177Arg variation was only detected in the proband but not in the affected offspring. Incomplete penetrance was suggested as an explanation for the presence of the variation in unaffected family members [30].

In this study, only one novel variation (c.491G>T, p.Arg164Leu) was detected in one of the 93 patients with high myopia. The protein information analysis using the SIFT program indicated that the p.Arg164Leu variation was unlikely to affect protein function, and the effect of this variation was similar to the effects seen in *Mus musculus* (p.Arg164His) or Zebrafish (p.Arg164Lys). However, it is impossible to confirm or deny the association of this variation with high myopia based on the current evidence, especially because of our limited understanding of complex diseases. In addition to the novel variant, a known c.803T>C variation was detected in 22 of the 93 patients as well as in 15 of the 48 controls. The genotype and allele frequencies of this variation were similar between the patients and controls (χ^2^ test, p=0.6175, and p=0.3529 separately). This variation was previously detected in ten Caucasian patients with high myopia as well as in 27 normal controls [30], suggesting that this variation is more likely to be a polymorphism.

*EPYC* (*DSPG3*), located in the mapping interval of MYP3, has been suggested as a candidate gene for high myopia [17]. An analysis of four single nucleotide polymorphisms (SNPs: rs1135866, rs1920748, rs1920751, and rs1920752) in *EPYC* was performed for 120 patients with high myopia and 137 controls, but no association was detected [31]. However, an evaluation of the coding and adjacent intronic sequences of *EPYC* has not reported in patients with high myopia thus far. In this study, no variation was detected in the sequences of five of the six coding and adjacent intronic regions of *EPYC* in 93 Chinese patients with high myopia. The PCR amplification of exon 5 of *EPYC* was unsuccessful despite the use of several primer pairs and different amplification conditions. An extremely high AT content in this region may be the cause of the difficulty with the PCR amplification.

In summary, our results suggest that both *OPTC* and *EPYC* are unlikely to play a major role in high myopia. Additional studies will be necessary to clarify these results.
